# Procalcitonin Levels Predict Acute Kidney Injury and Prognosis in Acute Pancreatitis: A Prospective Study

**DOI:** 10.1371/journal.pone.0082250

**Published:** 2013-12-13

**Authors:** Hua-Lan Huang, Xin Nie, Bei Cai, Jiang-Tao Tang, Yong He, Qiang Miao, Hao-Lan Song, Tong-Xing Luo, Bao-Xiu Gao, Lan-Lan Wang, Gui-Xing Li

**Affiliations:** Department of Laboratory Medicine, West China Hospital, Sichuan University, Chengdu, China; University of Sao Paulo Medical School, Brazil

## Abstract

**Background:**

Acute kidney injury (AKI) has been proposed as a leading cause of mortality for acute pancreatitis (AP) patients admitted to the intensive care unit (ICU). This study investigated the predictive value of procalcitonin (PCT) for AKI development and relevant prognosis in patients with AP, and compared PCT’s predictive power with that of other inflammation-related variables.

**Methods:**

Between January 2011 and March 2013, we enrolled 305 cases with acute pancreatitis admitted to ICU. Serum levels of PCT, serum amyloid A (SAA), interleukin-6 (IL-6), and C reactive protein (CRP) were determined on admission. Serum PCT was tested in patients who developed AKI on the day of AKI occurrence and on either day 28 after occurrence (for survivors) or on the day of death (for those who died within 28 days).

**Results:**

Serum PCT levels were 100-fold higher in the AKI group than in the non-AKI group on the day of ICU admission (*p*<0.05). The area under the receiver-operating characteristic (ROC) curve of PCT for predicting AKI was 0.986, which was superior to SAA, CRP, and IL-6 (*p*<0.05). ROC analysis revealed all variables tested had lower predictive performance for AKI prognosis. The average serum PCT level on day 28 (2.67 (0.89, 7.99) ng/ml) was significantly (*p*<0.0001) lower than on the day of AKI occurrence (43.71 (19.24,65.69) ng/ml) in survivors, but the serum PCT level on death (63.73 (34.22,94.30) ng/ml) was higher than on the day of AKI occurrence (37.55 (18.70,74.12) ng/ml) in non-survivors, although there was no significant difference between the two days in the latter group (*p* = 0.1365).

**Conclusion:**

Serum PCT is superior to CRP, IL-6, and SAA for predicting the development of AKI in patients with AP, and also can be used for dynamic evaluation of AKI prognosis.

## Background

Acute pancreatitis (AP) is a common illness with variable mortality and morbidity. Acute kidney injury (AKI) is a known severe complication of AP that increases AP’s mortality rate 5-fold (66.6% versus 14.5%) [1]. In current clinical practice, creatinine is still used as an indicator of kidney function. Serum creatinine (sCr) concentration does not change until about 50% of kidney function is lost [2], and varies with age, sex, muscle mass, medications, and hydration status [3]. The lag time between injury and loss of function means that reliance on sCr risks missing a therapeutic window, and may contribute to AKI’s high mortality rate. Furthermore, because there are few effective therapeutic options for treating AKI, it is imperative to discover markers for early prediction of AKI occurrence, as well as for assessing patients’ prognosis, in order to intervene earlier.

PCT is a 116-amino-acid precursor of the hormone calcitonin. In healthy individuals, It is formed in C-cells of the thyroid and degraded by specific proteases into calcitonin [4], limiting the amount of PCT entering the circulation. PCT plasma concentrations are therefore below 0.05 ng/mL in healthy humans [5]. However, during severe bacterial infections and sepsis plasma concentrations of PCT, produced mainly in in human peripheral blood mononuclear cells and modulated by bacterial lipopolysaccharides and sepsis-related cytokines [6], increase up to 10,000-fold (1 to 1000 ng/mL) [7]. PCT is recently put forward as a diagnostic marker of systemic bacterial infection and sepsis [8]–[10], which are the leading causes of acute kidney injury (AKI) in critically ill patients [11]–[14], and multiple studies [15]show that PCT levels are very high in patients with AP, and it has potential value in assessment of the severity and outcome of AP, which often are accompanied by translocation of lipopolysaccharide or other bacterial products from the gut to the systemic circulation [16]. In light of this, we aske whether PCT may be an early “biological” marker for AKI in patients with AP, as well as for prognosis for those patients who develop AKI.

## Methods

### Patients

Signed informed consents were obtained from all participants or their guardians, and the study was approved by the Ethics Committee of West China Hospital, Sichuan University. All the subjects involved in the research were patients who met criteria for AP according to the Atlanta classification and who were admitted to the ICU in West China Hospital within 72 hours after the onset of illness during the time period of January 2011 to March 2013. The classification of AP severity was based on a consensus conference held in Atlanta in 1992 [17]. AP is classified as severe if systemic and/or local complications are present. Each patient’s Ranson score at 48 hours after admission was calculated and recorded. Exclusion criteria were: (1) under 18 years of age; (2) absence of information on baseline renal function; (3) a history of nephrectomy, documented chronic kidney disease and renal transplantation; or (4) died or discharged within three days after admission. On admission, clinical data and blood samples for PCT, SAA, IL-6 and CRP concentrations were collected. Systemic inflammation response syndrome (SIRS) was defined as two or more of temperature <36°C or >38°C, heart rate >90 beats/min (bpm), white blood cell count <4×10^9^/L or >12×10^9^/L, and PaCO_2_<4.3 kPa.

Serum samples were collected from all included patients on three consecutive days after admission for routine renal function tests and to follow up the patients who met the criteria for AKI during that time. The patients with AKI were further grouped into survivors, defined as subjects who stayed alive for at least 28 days (≥28 days) after developing AKI, and non-survivors who died within 28 days (<28 days). Serum PCT levels were recorded for the AKI group on the day of AKI occurrence and on either day 28 after occurrence (for survivors) or on the day of death (for those who died within 28 days).

We diagnosed and classified AKI according to the RIFLE criteria, which categorizes the level of renal damage from AKI as risk, injury, failure, loss of function, and end-stage kidney disease [18]–[19], based only on patients’ creatinine levels (we were unable to collect full information on urinary output). Risk was defined as a ≥1.5-fold increase from baseline in serum creatinine within 48 h; injury was defined as a ≥2.0-fold increase in serum creatinine from baseline; failure was defined as either a ≥3.0-fold increase in serum creatinine from baseline or initiation of renal replacement therapy; loss of kidney function was defined as complete loss of kidney function for longer than four weeks; and end-stage kidney disease was defined as end-stage kidney disease lasting for longer than three months.

### Laboratory Test

Serum samples for PCT, SAA, CRP, IL-6, cystatin C, creatinine, and urea tests were obtained from unanticoagulated tubes by means of centrifugation at 3000 r/min for 15 minutes. PCT and IL-6 were determined via Elecsys- E170 (Roche Diagnostics, Germany), SAA via BNII (Siemens, Germany), CRP via Immage 800 (Beckman Coulter, USA), and cystatin C, creatinine, and urea via Modular-P800 (Roche Diagnostics, Germany).

### Data Analysis

Results for normally distributed variables are given as means±standard deviations (SD), and results for non-normally distributed variables are given as medians and interquartile ranges (IQR). Group comparisons used Student’s t test for normally distributed variables and non-parametric Mann-Whitney tests for non-normally distributed variables. Categorical variables were compared with a chi-square test. Two-sided statistical tests were used for all analyses, and differences between groups were considered to be significant at *p*<0.05. SPSS for Windows (version 13.0; Chicago, IL) was used to perform analyses. Receiver-operating curves (ROC) were generated to determine the cut-off values for optimal sensitivity and specificity for PCT, SAA, CRP, and IL-6, plotted by SigmaPlot 10.0 (Systat Software Inc, CA, USA). Their AUCs were compared using the Mann-Whitney U Test.

## Results

### Patient Characteristics

This clinical study included 305 patients with AP, 198 mild and 107 severe cases. A total of 52 patients (17% of the cohort, the AKI group) were diagnosed with AKI during the three-day follow up. Of these, 37 (71%) reached an “R” level of AKI severity, 10 (19%) an “I” level, and 5 (10%) an “F” level. On the basis of 28-day survival, the AKI patients were further divided into two sub-groups: survivors (33 cases) and non-survivors (19 cases). Table 1 shows the general condition of the patients within 24 h after admission. Of the included patients, 229 were male and 176 female, and the mean age was 49.9 years (range: 19–85). We found highly significant differences (*p*<0.001) in severity of pancreatitis, Ranson score, and mortality between the AKI and non-AKI groups: in the AKI group there were 8 mild and 44 severe panceatitis cases, the average Ranson score was 4.98±1.59, and mortality was 36.5%, whereas in the non- AKI group there were 190 mild and 63 severe panceatitis cases, the average Ranson score was 2.75±1.58, and mortality was 5.8%. There were no significant differences in average age, gender, urea, creatinine, cystatin C, SIRS score, or etiology of pancreatitis between the AKI and non-AKI groups.

**Table 1:** Initial characteristics of 305 patients with acute pancreatitis.

	Total (n = 305)	AKI (n = 52)	Non-AKI (n = 253)	P
Age (years)	49.9±16.0	50.9±15.9	48.6±16.2	0.50
Gender				0.06
Male	229	39	190	
Female	176	13	163	
Urea (mmol/L)	4.48 (3.45,7.48)	5.21 (3.75,7.84)	4.10 (3.15,5.47)	0.21
Creatinine (mmol/L)	49.4 (43.7,70.6)	57.3 (40.0,78.8)	49.4 (46.0,59.2)	0.79
Cystatin C (umol/L)	0.86 (0.69,1.02)	0.89 (0.72,1.15)	0.81 (0.68,0.91)	0.11
Severity of pancreatitis (n)				0.00
Mild	198	8	190	
Severe	107	44	63	
Ranson score	3.13±1.75	4.98±1.59	2.75±1.58	0.00
SIRS score	2.51±0.92	2.66±0.86	2.30±1.00	0.06
Respiration rate	26±7	28±8	24±5	0.01
Pulse	118±24	127±23	106±19	0.00
Temperature	37.8±1.0	37.9±1.2	37.7±0.7	0.34
WBC	13.9±8.5	12.7±8.0	15.5±9.0	0.12
Etiology of pancreatitis (n)				0.366
Gallstones	143	20	123	
Alcohol consumption	44	10	34	
Hyperlipemia	80	13	67	
Unknown	38	9	29	
Outcomes n (%)				0.00
Survivor	272	33 (63.5)	238 (94.2)	
Non-survivor	34	19 (36.5)	15 (5.8)	

± SD. Quantitative data of non-normal distribution are presented as median (25th and 75th percentiles). All data were collected within 24 h after admission. On the basis of 28-day survival, the patients were further divided into survivors and non-survivors. Qualitative data are presented as n. Quantitative data of normal distribution are presented as mean

= acute kidney injury; SIRS = Systemic inflammatory response syndrome; WBC = white blood cell count. AKI

### PCT, SAA, CRP, and IL-6 for Predicting AKI

**Figure 1:**
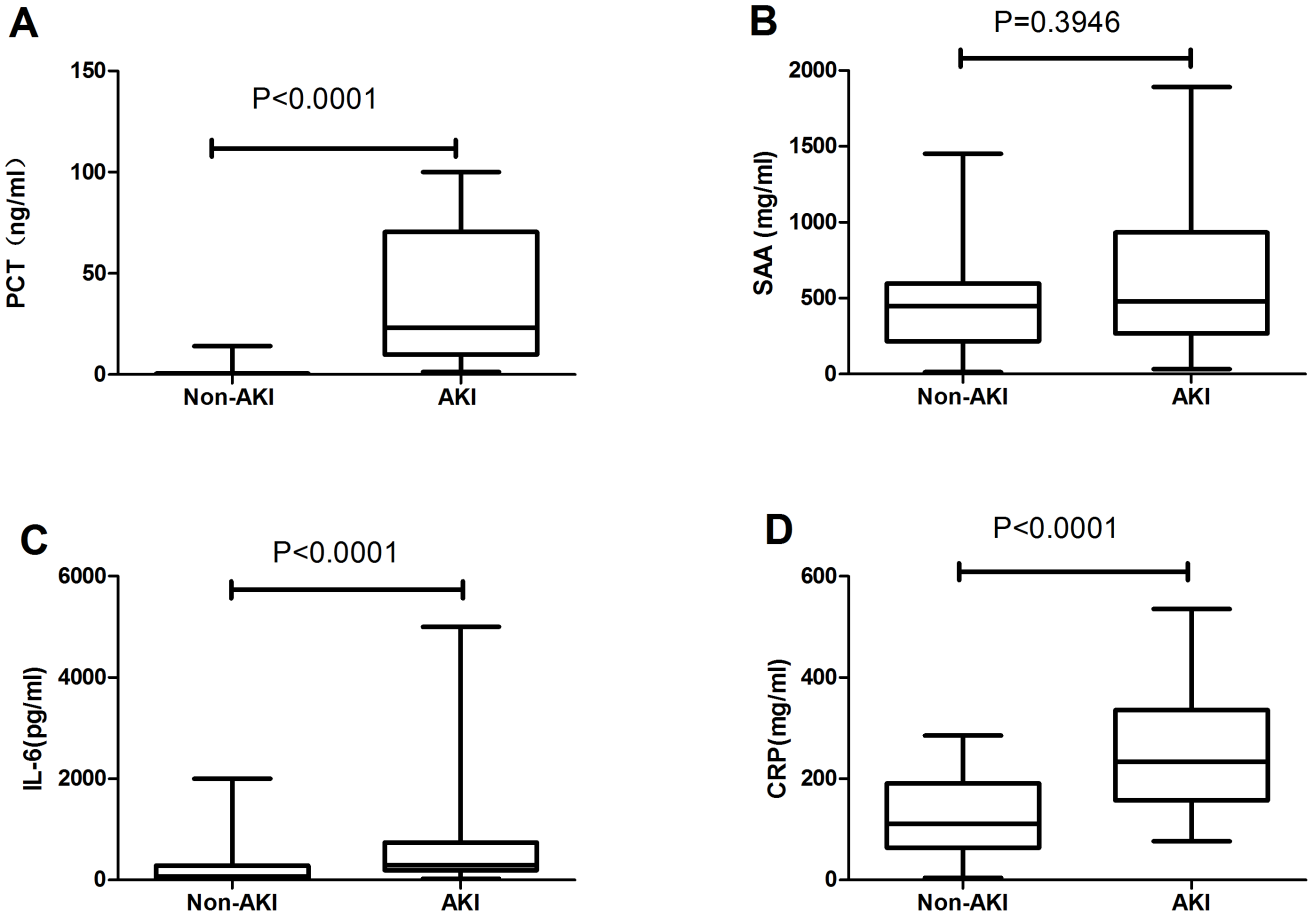
Initial levels of infection markers in non-AKI and AKI patients. The boxes indicate the interquartile range (IQR). The dark lines denote the median. The whiskers portray minimum and maximum values. Levels in the two groups were compared using the Wilcoxon rank sum test.

The AKI group and non-AKI groups had,highly significant differences (*p*<0.05) in PCT, CRP, and IL-6 serum concentrations on admission: the PCT levels were 100-fold higher in the AKI group, CRP was 2-fold higher, and IL-6 was 3.5-fold higher. However, no marked difference was observed in SAA levels between the two groups (Figure 1).

**Figure 2:**
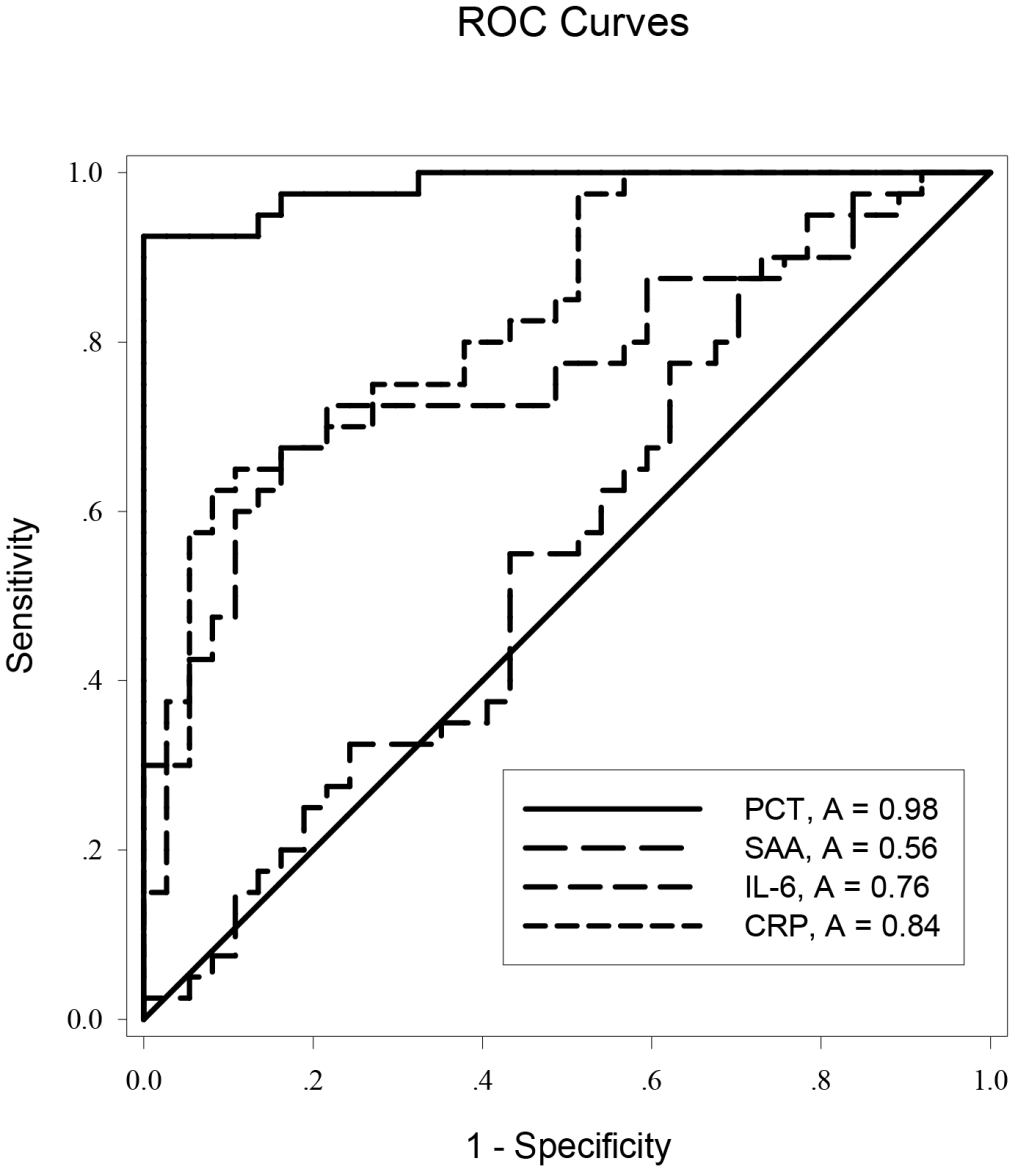
Receiver operating characteristic (ROC) curves for PCT, SAA, IL-6, and CRP for predicting AKI in AP.

**Figure 3:**
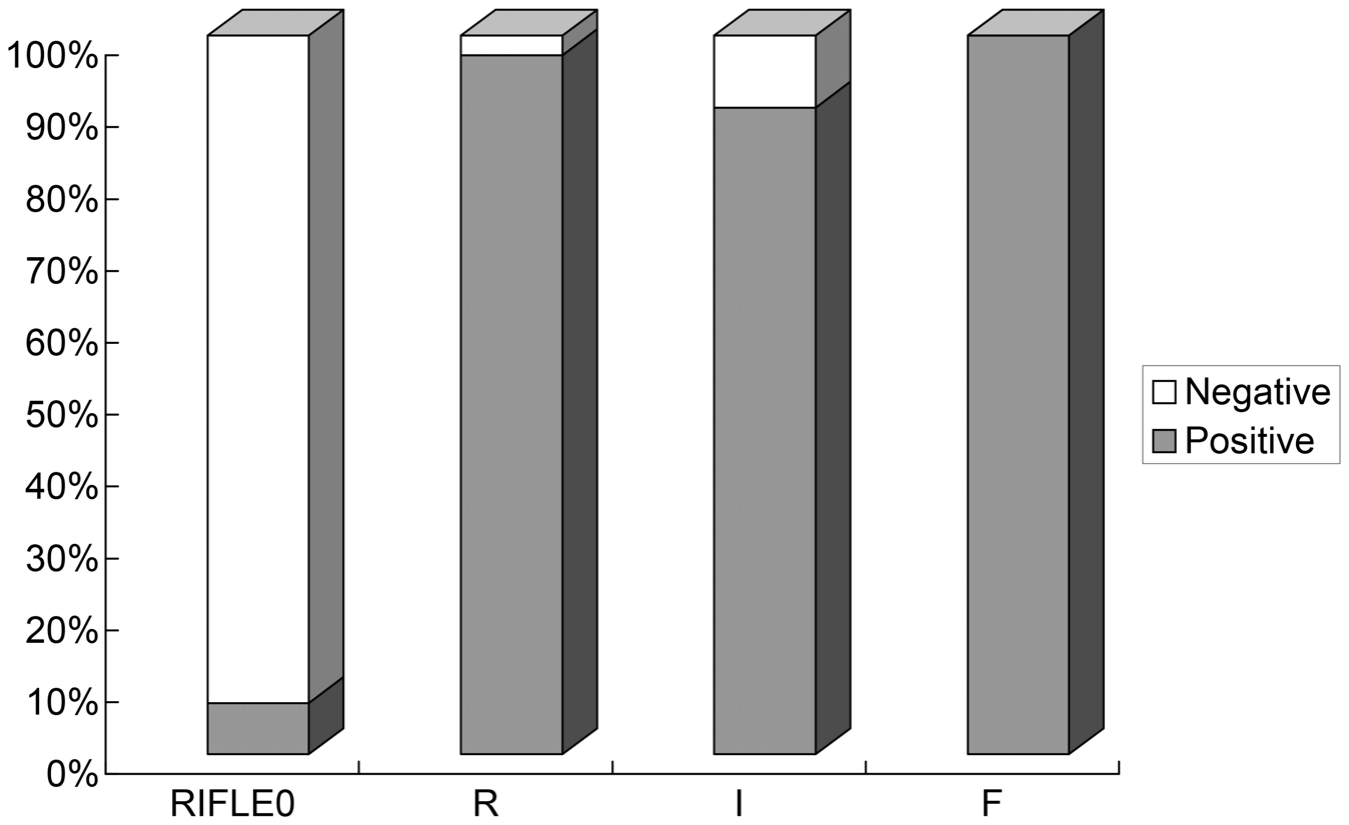
Relative percentage of patients with PCT levels above and below the cutoff value, stratified by RIFLE classes. The cutoff value for PCT levels was set at 3.30/ml. RIFLE = Risk, Injury, Failure, Loss, and End-stage kidney disease. P<0.001, Compared relative percentage between AKI(RIFLE class Risk, Injury, and Failure) and non-AKI(RIFLE 0) by chi-square test, and P = 0.832, among RIFLE class Risk, Injury, and Failure by the Cochran Armitage trend test.

**Figure 4:**
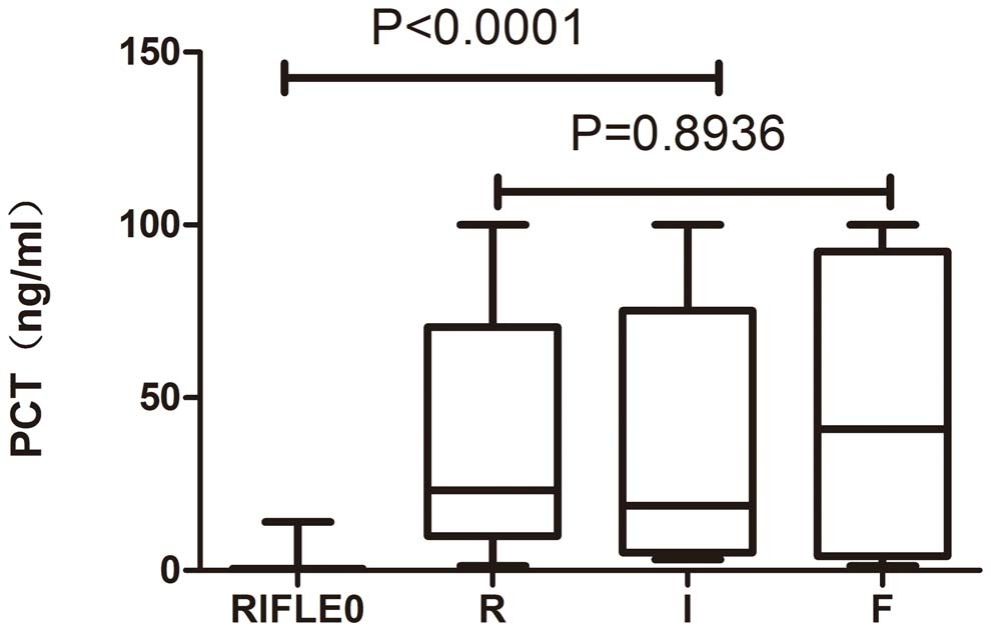
PCT levels in patients stratified by RIFLE classes. RIFLE = Risk, Injury, Failure, Loss, and End-stage kidney disease. PCT levels were compared between AKI (RIFLE classes Risk, Injury, and Failure) and non-AKI (RIFLE 0) patients using the Wilcoxon rank sum test, and PCT levels were compared among the RIFLE classes Risk, Injury, and Failure using the Kruskal-Wallis H test.

ROC curve analysis was applied to the initial values of the tested variables to assess their predictive performance. The area under the receiver-operating characteristic curves (AUC) (95% confidence interval (CI), p) of the initial levels of PCT, CRP, IL-6 and SAA for predicting the occurrence of AKI were 0.986 (0.966–1.000, *p*<0.05), 0.838 (0.738–0.919, *p*<0.05), 0.758 (0.647–0.869, *p*<0.05), and 0.547 (0.414–0.680, *p*>0.05), respectively (Figure 2). PCT was clearly (*p*<0.0001) superior to the others (Table 2). The cutoff value of PCT was 3.30 ng/ml, with a sensitivity of 97.2%, specificity 92.3%, positive likelihood ratio 12.62, negative likelihood ratio 0.03, and Youden’s index 0.87 (Table 3). But the initial level of PCT was unable to discriminate AKI severity: there was no significant difference among the “R”, “I”, and “F” classes in initial PCT levels (*p*>0.05; Figures 3 and 4).

**Table 2:** Differences in the areas under the ROC curves of PCT, SAA, CRP, and IL-6 for predicting AKI.

	PCT	SAA	CRP	IL-6
PCT	\	0.429(0.294,0.560)	0.147 (0.058,0.236)	0.221 (0.111,0.331)
		P<0.001*	P<0.001*	P<0.001*
SAA	\	\	0.28 (0.123,0.437)	0.206 (0.036,0.376)
			P<0.001*	P = 0.017*
CRP	\	\	\	0.074 (0.064,0.212)
				P = 0.293
IL-6	\	\	\	\

% confidence interval) between two indicators. * = P<0.05 according to the Mann-Whitney U Test. Results are presented as mean area difference (95

= procalcitonin; SAA = serum amyloid A; CRP =  C-reactive protein; IL-6 = interleukin-6. PCT

**Table 3:** Predictive performance comparison of PCT, SAA, CRP, and IL-6.

	PCT		SAA		CRP		IL-6	
	a	b	a	b	a	b	a	b
AUC	0.986*	0.559	0.557	0.506	0.838*	0.629	0.758*	0.445
Cutoff	3.30	52.02	766.0	1170.1	124.5	294.5	144.91	641.03
Sensitivity	97.2%	39.1%	29.7%	22.7%	91.9%	55.2%	83.8%	32.1%
Specificity	92.3%	85.7%	87.5%	86.7%	62.5%	87.0%	67.5%	73.0%
LR^+^	12.62	2.74	2.38	1.71	2.45	4.09	2.57	1.19
LR^–^	0.03	0.71	0.80	0.89	0.13	0.52	0.24	0.93
γ	0.87	0.25	0.17	0.09	0.54	0.42	0.51	0.05

* = P<0.05 compared with the AUC of the reference line in the ROC analysis.^a^ ROC curve analysis was used to distinguish between AKI and non-AKI. b. ROC curve analysis was used to distinguish between survivors and non-survivors.

= procalcitonin; SAA = serum amyloid A; CRP = C-reactive protein; IL-6 = interleukin-6; AUC = area under the ROC curve; LR^+^ = positive likelihood ratio; LR^–^ = negative likelihood ratio; γ = Youden’s index. PCT

### Evaluation of AKI Prognosis

**Figure 5:**
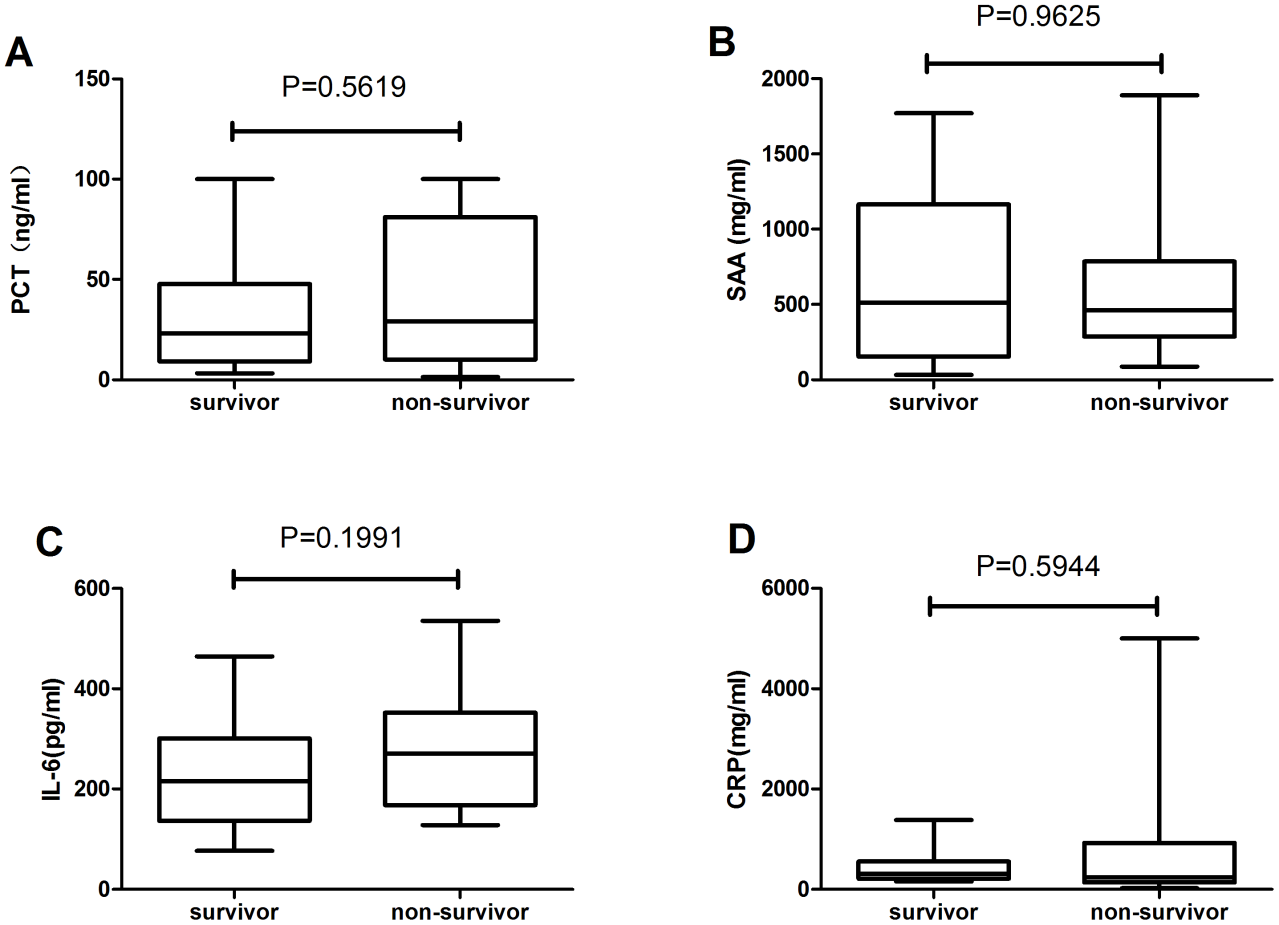
Initial levels of infective markers between AKI survivors and non-survivors. The boxes indicate the interquartile range (IQR). The dark lines denote the median. The whiskers indicate minimum and maximum values. Marker levels were compared between the two groups using the Wilcoxon rank sum test.

When comparing the survivor and non-survivor groups of AKI patients, we found that no marked differences were observed in PCT,SAA, IL-6, or CRP levels between the two groups (Figure 5).

**Figure 6:**
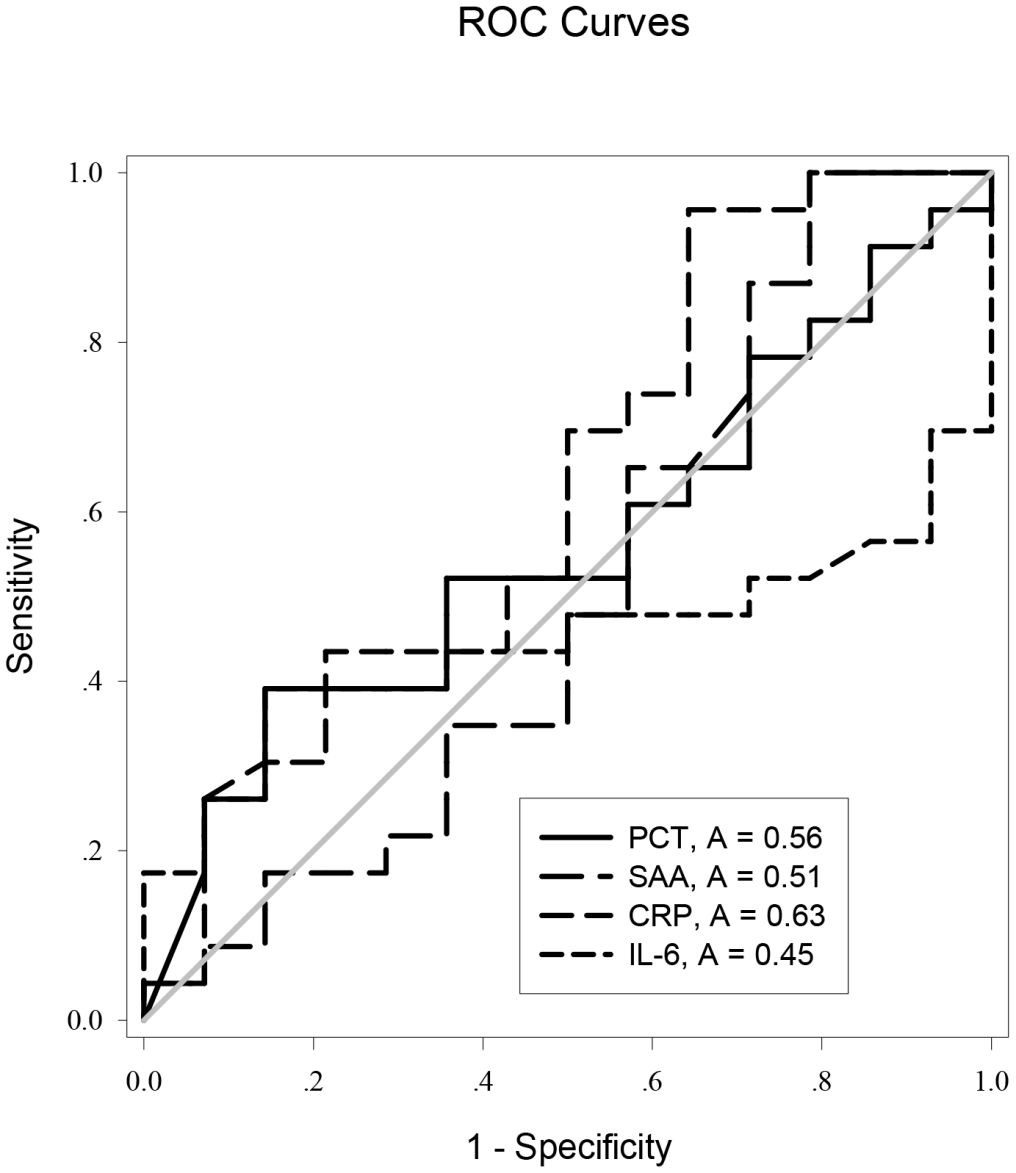
Receiver operating characteristic (ROC) curves for PCT, SAA, IL-6, and CRP for AKI prognosis.

ROC curves were generated for these variables to evaluate their performance for disease prognosis. The AUC (95% confidence interval (CI), *p*) of the initial levels of PCT, CRP, IL-6, and SAA for AKI prognosis were 0.559 (0.368–0.750, *p* = 0.552), 0.629 (0.432–0.826, *p* = 0.194), 0.446 (0.256–0.635, *p* = 0.583), and 0.506 (0.293–0.718, *p* = 0.95), respectively (Figure 6). The cutoff value for PCT was 28.32 ng/ml, with a sensitivity of 39.1%, specificity 85.7%, positive likelihood ratio 2.74, negative likelihood ratio 0.71, and Youden index 0.25 (Table 3).

**Figure 7:**
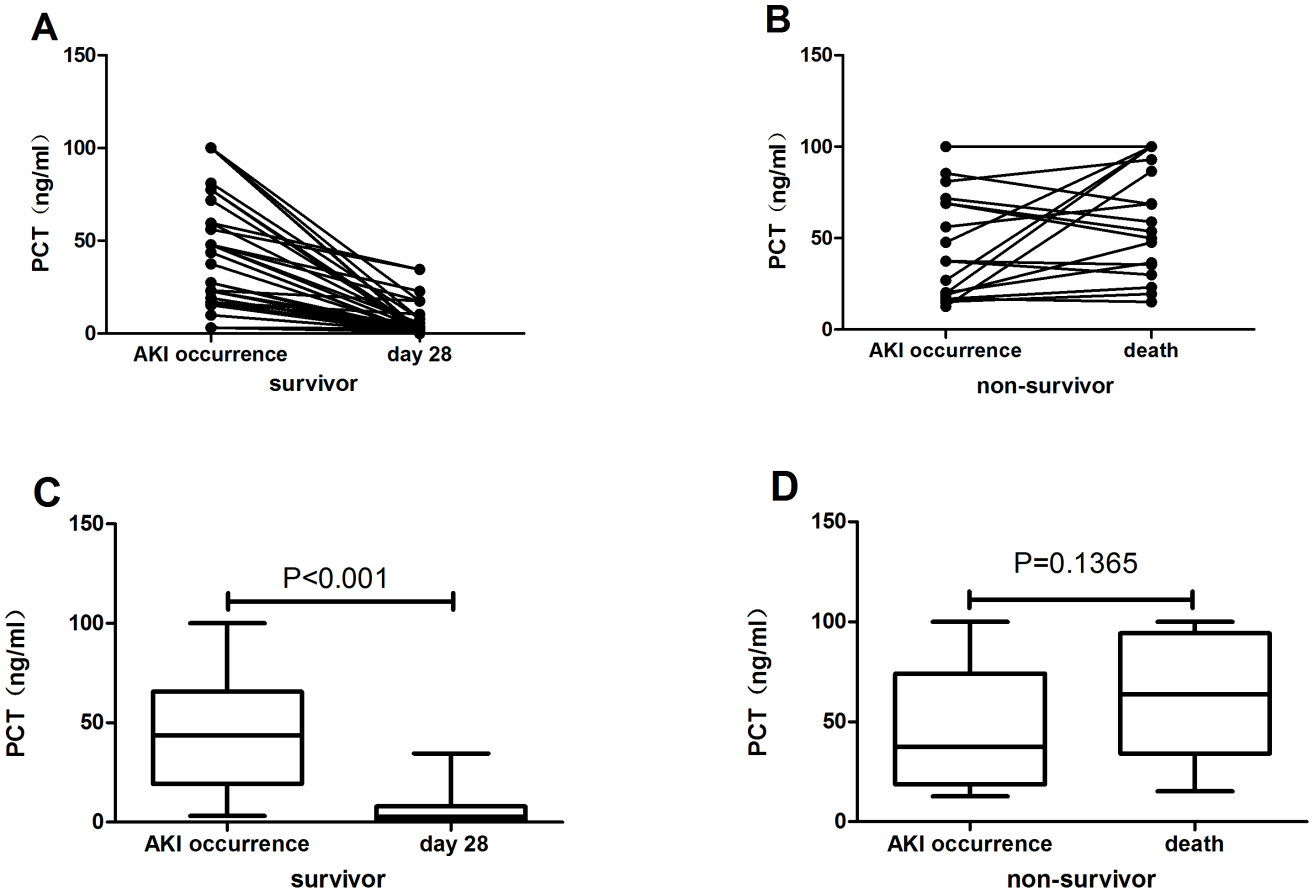
Figure 7. Relationship between serum PCT levels and prognosis. **A.** The trend of serum PCT levels in survivors during the period from AKI occurrence to termination of follow up. **B.** The trend of PCT levels in non-survivors during the period from AKI occurrence to death. **C.** Serum PCT levels on the days of AKI occurrence and of termination of follow-up in survivors. **D.** Serum PCT levels on the days of AKI occurrence and of death in non-survivors. PCT levels of the two days were each compared using the Wilcoxon signed-rank test.

The PCT levels of most of the survivors had significantly decreased by day 28, whereas there was no decline among the non-survivors between admission and the day of death (Figure 7A, B, C, D). Thus, the trend in serum PCT levels was in concordance with AKI prognosis.

## Discussion

We sought to determine Whether PCT has a role in predicting AKI occurrence and its prognosis in AP patients. Our results indicate that serum PCT level can be an early, sensitive, specific biomarker for predicting AKI in patients with AP, with an ideal cutoff value of 3.30 ng/ml, and that changes in serum PCT levels during the course of the disease may assist in assessing patients’ prognosis.

AKI in the setting of AP was associated with a 10-fold increase in mortality (74.7% versus 7%) in a study of 563 patients [20]. A similar result was reported in another study, showing 71.2% mortality versus 6.8% in severe acute pancreatitis (SAP) patients with and without AKI [21]. Our study found the severity of pancreatitis in the AKI group was significantly higher than in the non-AKI group on admission and there was a 6-fold higher mortality rate (36.5% with AKI versus 5.8% without AKI) in AP patients, which suggests that SAP itself is associated with higher incidence of AKI, and that both SAP and AKI may be the factors that increased patients’ mortality risk.

Several studies have found that the traditional indicators of renal function have little value for early prediction of AKI [22]–[24]. In the present study, we did not find elevated serum urea, creatinine, or cystatin C levels in the AKI group at enrollment. Rather, most of the patients who later developed AKI displayed significantly elevated serum levels of PCT, CRP, and IL-6, the main indicators of inflammation. This indicates that inflammation was closely correlated with AKI occurrence in AP patients, which is consistent with the currently dominant view [25]–[30] that sepsis and SIRS are among the main causes of AKI.

Our ROC analysis determined that of PCT, SAA, CRP, and IL-6, PCT had the best accuracy in predicting AKI in patients with AP, with an AUC of 0.986. The reason for PCT’s utility as a predictive marker is an important consideration. Several studies [8]–[10] have found that elevated PCT levels in human blood can be detected in cases of sepsis and bacterial infection, and PCT has been described as a new and innovative parameter of sepsis. It has also been reported [31], [32] that elevated PCT levels correlate with the severity and clinical outcome of sepsis, including organ dysfunction, such as renal injury. We simultaneously conducted another study [33] of 1361 patients with suspected of infections, mainly pulmonary and intra-abdominal, and the ROC curve showed that the AUC of PCT for predicting AKI in those patients was 0.830. The study found that PCT can be used as a predictive marker for sepsis-induced acute kidney injury in patients with symptoms of infection. Wagner KE et al [34] and Martinez JM et al [35], based on a prorcine sepsis model, found that both early and late immuno-neutralization of PCT significantly ameliorated systemic and renal complications. Furthermore, Araujo M,et,al [36] conducted an experiment to investigated the direct effect of PCT on mesangial cells, and showed that PCT had direct cytotoxic properties. Thus, PCT may play as a toxic mediator in sepsis-related AKI. In healthy people, the normal intestinal mucosa forms an effective barrier to intraluminal bacteria and toxins, while in AP patients, gastrointestinal mucosal damage takes place in the early phase of AP as a result of local or systemic inflammation response, hypovolemic shock, and gut ischemia [37]–[41]. Intestinal flora enter into circulation following dysfunction of the gut barrier, which is considered to be the main mechanism leading to secondary infection or sepsis in pancreatitis patients [39]–[41]. Therefore, it is not surprising that PCT is an early predictor of AKI occurrence in patients with AP. Our results also suggest that infection or sepsis may be a risk factor for AKI occurrence in AP patients.

The results of the present study also suggest that CRP and IL-6 have some predictive value for AKI in AP patients. However, these two indicators can significantly increase in response to stress, and are affected by a variety of infectious and non-infectious factors [42]–[45], so they lack specificity. We found that their sensitivities, specificities, positive likelihood ratios, negative likelihood ratios, and Youden’s indices were poorer than those of PCT, and that PCT used alone had good performance in predicting AKI. So to predict AKI in AP patients, we recommended measuring only serum PCT levels rather than simultaneously determining other inflammatory markers as well.

We found that serum PCT level on admission was not able to discriminate AKI severity, and ROC analysis revealed that the initial serum levels of all variables also performed poorly in determining AKI prognosis. The PCT levels of most of the AKI survivors had significantly decreased by day 28, whereas there was a slight but non-significant increase in PCT levels in non-survivors between admission and the day of death. Our results suggest that changes in serum PCT levels during the course of the disease may assist physicians in identifying patients at risk for deterioration and progression. Some studies [46], [47] have similarly found that If PCT levels decrease by more than 30% within the first 24 h of initiating antibacterial treatment, it indicates that the treatment is appropriate and the infection is under control; if PCT levels increase, it means that the antimicrobial treatment must be changed; and if PCT levels continuously increase, the host response to infection is very poor and the patient’s condition is likely to deteriorate. Thus, changes in PCT level could be used to assess patients’ prognosis during treatment for AKI and other infection-related conditions.

In order to more fully understand the relationship between changes in PCT levels and patents’ prognoses, serial measurements of serum PCT levels will be needed. One limitation of our study was that we only determined PCT levels at two time points. In addition, there are many possible causes for mortality among AP patients with AKI, some of them unrelated to PCT levels, and our study did not adjust for this because the sample size was not large enough for us to perform a further analysis of causality or to adjust for additional influencing factors. This is a potentially important reason for the low performance of initial PCT level in determining AKI prognosis. More studies with larger sample sizes are needed to confirm PCT’s utility.
